# Special Issue “Electric, Magnetic, and Electromagnetic Fields in Biology and Medicine: From Mechanisms to Biomedical Applications: 2nd Edition”

**DOI:** 10.3390/bioengineering12070739

**Published:** 2025-07-07

**Authors:** Stefania Romeo, Anna Sannino

**Affiliations:** CNR—Institute for Electromagnetic Sensing of the Environment (IREA), 80124 Napoli, Italy

## 1. Introduction

Electric, magnetic, and electromagnetic fields (EMFs) are widely used in everyday life, as well as in specific occupational environments and clinical settings. EMF-based technologies employ different parts of the spectrum, from static fields to low- and high-frequency EMFs encompassing millimeter waves and THz [1].

Exposure to these fields raises concerns about the possible effects on human health. On the other hand, biomedical applications of non-ionizing radiation are successfully employed for diagnosis and therapy (e.g., electroporation-based treatments, microwave hyperthermia, transcranial magnetic stimulation, etc.). There is great interest in evaluating the associated interaction mechanisms, which are also relevant in fostering the development of new biomedical applications and the optimization of the existing ones.

This Special Issue, entitled “Electric, Magnetic, and Electromagnetic Fields in Biology and Medicine: From Mechanisms to Biomedical Applications: 2nd Edition”, includes contributions focusing primarily on the therapeutic and diagnostic applications of EMFs. In vitro, in silico, and human studies are presented where the aim was either to optimize technical aspects of the applications, or to provide insight into biological, biophysical, electrical, or electrochemical mechanisms. Overall, these contributions present an overview of the broad spectrum of established and potential applications of electromagnetics in the biomedical field.

## 2. Contributions to the Special Issue

The call for articles on “Electric, Magnetic, and Electromagnetic Fields in Biology and Medicine: From Mechanisms to Biomedical Applications: 2nd Edition” resulted in a total of 14 accepted manuscripts: 12 regular papers, and 2 review papers. A brief description of each contribution is reported in the following passages, with the papers organized based on the topics addressed.

### 2.1. Transcranial Magnetic and Direct Current Stimulation

Transcranial Magnetic and Direct Current Stimulation (TMS, tDCS) are non-invasive brain stimulation techniques that have gained increased interest in recent decades not only for their potential application in the treatment of mental health conditions (addiction, depression, anxiety, etc.), but also for other neurological conditions as well as for rehabilitation purposes. TMS uses coil-generated magnetic fields to induce an electrical current in the brain and stimulate specific cortical regions, whereas tDCS applies low-intensity direct currents by means of suitable electrodes to modulate brain excitability through changes in the resting membrane potentials [2].

In this Special Issue, five full papers addressed TMS or tDCS, considering different aspects of the techniques and their applications.

Pantovic and co-workers performed a double-blind, randomized, between-subject, sham-controlled, experimental study to analyze whether cerebellar tDCS could improve motor learning in a complex overhand throwing task in thirty young adults. The subjects were able to improve accuracy in performing the task, but there were no significant differences between those subjected to tDCS and sham controls. The authors concluded that tDCS failed to improve motor learning in this complex motor task to a greater degree than practice alone (sham) in the experimental conditions tested in the study, and that future studies are needed to fully determine the efficacy of cerebellar tDCS for potentially enhancing motor skill acquisition and learning in healthy young adults [3].

Pantovic and co-workers addressed the optimization of Intracortical Facilitation (ICF), which is a paired-pulse TMS measurement used to quantify interneuron activity in the primary motor cortex in healthy populations and motor disorders. Specifically, they considered the role of time between ICF trials (inter-trial interval; ITI). In a within-subject experimental design, twenty young adults participated in an experimental session involving voluntary muscle contraction under four separate ICF trial blocks, with each utilizing different ITIs (4, 6, 8, and 10 s). The outcome was assessed by analyzing the electromyographic response. The main finding of the study was that ICF values were similar for all four ITIs tested and did not significantly change over the course of time for any of the ICF blocks considered [4].

In a similar study, de Albuquerque and co-authors assessed the role of ITIs on Short-Interval Intracortical Inhibition (SICI), which is a common paired-pulse TMS measure of the primary motor cortex interneuron activity in healthy subjects and neurological disorders. The experiments were performed on the right-hand of twenty-three healthy, young participants, and involved voluntary muscle contraction under four SICI trial blocks, each utilizing different ITIs (4, 6, 8, and 10 s). The outcome was assessed by analyzing the electromyographic response. The main findings indicated that measurements of SICI neither differed between ITIs (ranging between 4 and 10 s) nor demonstrated significant time-dependent amplitude changes within blocks of trials [5].

In another paper, Robins and co-workers addressed an alternative approach to magnetic neurostimulation in which the time-varying magnetic field (and the resultant, induced electric field) is generated not by current-carrying coils, but by using rotating permanent magnets. The authors assessed the electric field characteristics of various rotating magnet configurations through computational modeling and validated the results via experimental measurements of field strengths on a head phantom. The results of the analysis showed that the maximum induced E-field strength on the head surface was around 0.1% of the field strength induced by conventional TMS, and that electric field strength depended on rotational frequency. Further research is needed to conduct simulations of rotating magnetic stimulation on anatomically accurate head models, as well as to optimize treatment parameters such as stimulation frequency and magnet placement [6].

In the paper by Camera and co-authors, low-frequency numerical dosimetry approaches used for TMS studies were compared across simplified and realistic anatomical models to assess their accuracy in evaluating induced electric fields. For the study, a typical figure-of-8 coil was used as the TMS source, and the performance levels of two simulation platforms, SimNIBS v.4.0.0 [7] and Sim4Life v7.2.4 (ZMT, Zurich MedTech, Zurich, Switzerland), were compared based on three different exposure scenarios: a homogeneous sphere, a sphere with an internal discontinuity, and a head model derived from Magnetic Resonance Imaging (MRI) data. The results indicated that the differences between the obtained results were larger upon increasing the geometric complexity of the model. However, the differences remained contained overall [8].

### 2.2. Mechanisms and Clinical Applications of Pulsed Electromagnetic and Electric Fields

Pulsed Electromagnetic (PEMFs) and Electric Fields (PEFs) are successfully employed within numerous medical applications, including the treatment of musculoskeletal disorders, like non-union fractures, osteoarthritis and osteoporosis, and also the stimulation of bone healing, promotion of wound healing, electrical stimulation of tissues, and can even be used in adjuvant cancer treatments. Nevertheless, a clear understanding of the underlying molecular mechanisms and associated robust clinical outcomes remains elusive because of their diverse use [1,9].

In this Special Issue, three full papers and two reviews addressed the biological mechanisms and clinical applications of PEMFs and PEFs.

Costantini and co-authors assessed the inflammatory, antioxidant, cell proliferation, and wound healing response of human primary dermal fibroblasts (HDFs) isolated from normal and ulcerated areas of venous leg ulcer patients and then exposed to PEMFs in the radiofrequency range by means of a commercial medical device. The exposure to RF PEMFs induced an earlier reduction in the scratch-induced cell-free area displayed by exposed ulcer-HDFs compared to the unexposed ones and even to normal-HDFs. This trend persisted after 24 h, suggesting an increase in the repair ability in PRF-EMF-exposed ulcer-derived HDFs. The results of the study show that a PEMF may affect ulcer-HDF cell proliferation and modulate the expression and production of cytokines, leading to an improvement in wound healing by activating the robust migration of fibroblasts and by further stimulating the inflammatory response [10].

Sun and co-workers analyzed whether biphasic, charge-balanced electric impulses, generated with either manual calibration, capacitive electrode coupling, or feedback regulation of electrode polarization, could reduce the electrochemical reactions at the interface of graphite electrodes used for the continuous stimulation of myocardial tissue. Faradaic reactions at the electrode surface were quantified using phenol red as a redox-sensitive tracer. The study demonstrated that charge control is an effective measure to improve the electrochemical compatibility of biphasic electrical impulses, whereas the capacitive coupling approach gave less satisfactory results. Further studies are thus warranted to understand the biological implications of this technique [11].

In Asadipour et al., the authors analyzed the effects of post-pulse waveform nanosecond (ns) PEFs, i.e., low-intensity, spurious pulses occurring after the main one due to an incomplete discharge, that have been demonstrated to affect the biological effects of nsPEFs. Two commonly used pulse generator designs, both featuring identical main pulse characteristics but different post-pulse shapes, were used to compare the effects on various cellular endpoints. The thresholds for the dissipation of the mitochondrial membrane potential, loss of viability, and increase in plasma membrane permeability all occurred at different pulsing numbers for the two generators, and biphasic effects were detected in only one case. The paper demonstrated that conditions resulting from low post-pulse intensity charging have a significant impact on cell responses and should be considered when comparing the results from similar pulse waveforms [12].

The first review, presented by A. Szasz, reports on modulated electro-hyperthermia (mEHT), a variation of the conventional hyperthermia treatment, which selectively heats malignant tissues and makes them more sensitive to oncological treatments. Specifically, the author discusses pulsed mEHT, in which heat is applied to tumor tissue in short, controlled bursts rather than continuously. This approach can potentially enhance the effectiveness of cancer treatments while minimizing the damage to healthy surrounding tissues [13].

In the second review, presented by Kaadan and co-workers, scientific literature regarding the use of PEMFs for the treatment of fresh fractures, delayed union, and non-union, and possible underlying mechanisms, was discussed. The review describes biological pathways behind the bone-repair effect of PEMFs, starting from the cellular scale, and continuing up to the tissue and organismal scale. Overall, the use of PEMFs in orthopedic applications could potentially become a standard adjunctive therapy in the management of fractures and non-union thanks to the safety profile, absence of adverse effects reported, and non-invasive nature, provided that a better understanding of the mechanisms is unlocked [14].

### 2.3. Magnetic Resonance Imaging

MRI is a non-invasive imaging modality that uses intense, static magnetic fields and RF pulses to generate detailed images of internal body structures. MRI has become a cornerstone in medical diagnostics due to its high spatial resolution and excellent contrast resolution, especially for soft tissue. MRI is widely used in neurology, cardiology, musculoskeletal imaging, and oncology, among other fields, providing detailed information that can aid in diagnosis, treatment planning, and monitoring. Its advantages include high-contrast resolution, non-ionizing radiation, and the ability to acquire multiplanar images. On the other hand, limitations include long scan times, high costs, contraindications in patients with certain implants or devices, and sensitivity to patient motion, although the MRI technology continues to expand in clinical applications [15].

This Special Issue includes two full articles addressing this topic.

Guo et al. focused on advanced diagnostic techniques to improve the visualization of biological tissues with specific proton relaxation characteristics. They investigated whether the use of imaging techniques such as Zero Echo Time (ZTE) and the Ultrashort Echo Time (UTE) sequence can directly detect collagen protons in bone and tendons in comparison to water protons. Their main conclusions are that the ZTE sequence, like the UTE one, cannot directly detect collagen protons in bone and tendons, as the MRI signal originates from water protons and not via the collagen matrix. These results underscore the limits of current MRI techniques for direct collagen imaging, and the need for alternative imaging techniques or biochemical markers to study collagen integrity in bone and tendons [16].

Wang and co-workers addressed the study of electromagnetic fields and the dielectric properties of human tissues in the context of Ultra-High-Field Magnetic Resonance Imaging (UHF MRI), such as 7 T systems, with a focus on managing safety related to the Specific Absorption Rate (SAR). Their research proposes a computational framework based on High-Dimensional Model Representation (HDMR) as an effective alternative to traditional methods, and the proposed modeling framework provides an accurate, computationally efficient method for SAR estimation while reducing computational costs [17].

### 2.4. Other Topics

The use of magnetic scaffolds (MagSs) represents a fascinating and rapidly evolving area in biomedical engineering, holding significant promise for both tissue repair and cancer treatment. The integration of magnetic nanoparticles (MNPs) into biocompatible scaffold materials is expected to facilitate remote manipulation and localized effects using external magnetic fields. Overcoming these challenges will require new interdisciplinary efforts and technological advances, including the development of mathematical tools and additional elaborations to ensure the biocompatibility of MNPs [18]. From this perspective, Lodi et al. assessed the performance of MagSs, which are biomaterials combined with MNPs for drug delivery (DD) in tissue engineering (TE) and cancer therapy (CT). The use of MagSs is discussed as an innovative system for controlled drug release and tissue repair, using static or dynamic magnetic stimuli. The authors analyzed MagS drug release literature data and fitted them to mathematical and computational models. The study establishes a strong quantitative foundation for MagS-based DD, aiding future research in TE and CT applications. Future work should focus on improving MagS formulations, optimizing magnetic properties, and integrating advanced modeling techniques for better predictability and efficiency [19].

A further contribution to this Special Issue discussed the use of microwaves in biomedical applications. In recent years, microwave energy has been successfully exploited within medicine to treat diseases such as cancer and microbial infections via ablation therapy and for rapid cell lysis. [20]. Moreover, microwaves can be used to enhance electrochemical biosensor performance; for example, they can help modify electrode surfaces or facilitate rapid chemical reactions, increasing the sensitivity and response speed of the biosensor. In some cases, microwaves are also used to activate or boost electrochemical reactions, making biosensors more efficient and suitable for quickly detecting small amounts of biomolecules or pathogens [21].

In this framework, Joshi et al. presented a novel method for the rapid detection of *Clostridioides difficile* (*C. difficile*) spores in stool samples. The study introduces a microwave-enhanced lysis approach for DNA extraction combined with electrochemical biosensing to identify *C. difficile* toxin genes. A custom-built microwave cavity operating at 2.45 GHz was used to lyse *C. difficile* spores within 5 s. The microwave-enhanced method significantly reduces the time needed for *C. difficile* detection (<10 min) compared to traditional methods. The study introduces a diagnostic tool for quickly and accurately detecting *C. difficile* infections as an alternative to existing diagnostic tests [22].

## 3. Conclusions

In the first edition of the Special Issue on “Electric, Magnetic, and Electromagnetic Fields in Biology and Medicine: From Mechanisms to Biomedical Applications”, the included contributions mainly addressed EMF exposure assessment, the biological effects of EMF exposure, and health risk evaluation [23].

In the second edition, the included contributions mainly address the biomedical applications of non-ionizing radiation, with different approaches spanning from in vitro and human studies, to numerical modeling for the optimization of diagnostic or therapeutic techniques, as well as to improve specific, technical aspects related to the development of EMF-based technologies.

Overall, the papers presented in this Special Issue represent a diverse account of EMF-based application complexity, guiding the reader through explanations of general problems related to the use of EMFs and the basic results obtained from experimental and in silico studies. We hope readers will find these articles useful and informative and inspire further ground-breaking research in this area.

## Abbreviations

The following abbreviations are used in this manuscript:

*C. difficile*	*Clostridioides difficile*
CT	Cancer Therapy
DD	Drug Delivery
HDF	Human Dermal Fibroblasts
HDMR	High-Dimensional Model Representation
ICF	Intracortical Facilitation
ITI	Inter-Trial Interval
MagS	Magnetic scaffold
mEHT	Modulated Electro-HyperThermia
MNP	Magnetic Nanoparticle
MRI	Magnetic Resonance Imaging
ns	Nanosecond
PEF	Pulsed Electric Field
PEMF	Pulsed ElectroMagnetic Field
RF	Radiofrequency
SAR	Specific Absorption Rate
SICI	Short-Interval Intracortical Inhibition
tDCS	Transcranial Direct Current Stimulation
TE	Tissue Engineering
TMS	Transcranial Magnetic Stimulation
UHF	Ultra-High-Field
UTE	Ultrashort Echo Time
ZTE	Zero Echo Time
